# On the heterogeneity of human populations as reflected by mortality dynamics

**DOI:** 10.18632/aging.101112

**Published:** 2016-11-22

**Authors:** Demetris Avraam, Séverine Arnold, Olga Vasieva, Bakhtier Vasiev

**Affiliations:** ^1^ School of Social and Community Medicine, University of Bristol, Bristol, UK; ^2^ Department of Actuarial Science, Faculty of Business and Economics (HEC Lausanne), University of Lausanne, Lausanne, Switzerland; ^3^ Institute of Integrative Biology, University of Liverpool, Liverpool, UK; ^4^ Department of Mathematical Sciences, University of Liverpool, Liverpool, UK

**Keywords:** mortality dynamics, mathematical modeling, model fitting, demography, population genetics

## Abstract

The heterogeneity of populations is used to explain the variability of mortality rates across the lifespan and their deviations from an exponential growth at young and very old ages. A mathematical model that combines the heterogeneity with the assumption that the mortality of each constituent subpopulation increases exponentially with age, has been shown to successfully reproduce the entire mortality pattern across the lifespan and its evolution over time. In this work we aim to show that the heterogeneity is not only a convenient consideration for fitting mortality data but is indeed the actual structure of the population as reflected by the mortality dynamics over age and time. In particular, we show that the model of heterogeneous population fits mortality data better than other commonly used mortality models. This was demonstrated using cohort data taken for the entire lifespan as well as for only old ages. Also, we show that the model can reproduce seemingly contradicting observations in late-life mortality dynamics. Finally, we show that the homogenisation of a population, observed by fitting the model to actual data of consecutive periods, can be associated with the evolution of allele frequencies if the heterogeneity is assumed to reflect the genetic variations within the population.

## INTRODUCTION

One of the central problems in ageing-related studies is understanding the processes which underlie senescence and revealing the biological functions which deteriorate in organisms over the course of their lifespan. Ageing is associated with the acceleration of mortality expressed by the exponential increase of mortality rate over age, as described by the Gompertz law. The Gompertz law represents a fundamental mortality law and was verified by demographic observations across different countries, different time periods, and even different species [1]. The analysis of available data on mortality rates for various diseases also indicates that for most diseases there is a considerably wide age range where the mortality rate also increases exponentially [2, 3].

The exponential growth of mortality is not observed at young (before sexual maturity) and extremely old ages. Many researchers consider the exponential law of mortality to be “natural” while the deviations from it need to be explained. Initial mortality at age zero is high due to the noticeable proportion of deaths amongst neonates. The infant and child mortality rate is lower than the initial mortality at age zero and reaches its lowest level roughly at age 10. During teenage and young-adult ages (10-35 years) the mortality trajectory produces a peak which is associated with accidental (i.e. due to external and other non-biological causes) mortality. After the reproductive period, in which the accidental hump arises, the mortality rate advances exponentially up to very old ages when it slows down.

The observation that the exponential law of mortality does not apply at older ages was first made by Gompertz. In his 1825 paper, Gompertz stated that “*The near approximation in old age, according to some tables of mortality, leads to an observation, that if the law of mortality were accurately such that after a certain age the number of living corresponding to ages increasing in arithmetical progression, decreased in geometrical progression, it would follow that life annuities, for all ages beyond that period, were of equal value; for if the ratio of the number of persons living from one year to the other be constantly the same, the chance of a person at any proposed age living to a given number of years would be the same, whatever that age might be;*” recognising that the probability of surviving (and consequently, the mortality rate) levels-off at extremely old ages.

The divergence of mortality from its exponential increase at extremely old ages is generally accepted as valid by the majority of biologists and bio-demographers [4, 5] although some data do not support this observation (see for example [6–8]). Furthermore, existing data on mortality dynamics at advanced ages are so controversial [5] that up-to-date performed studies have not given a definite answer on what mathematical function (logistic, quadratic, etc.) can describe the data at those ages [9–11].

The absence of a definite explanation for the trend of mortality at very old ages renders the analysis of “oldest-old” mortality as an essential area of research in demography. Analysing mortality data for old ages is challenging as such data are usually unreliable and often statistically noisy as the number of survivors at those ages is small. The stochastic effects at very old ages are often seen as fluctuations in mortality dynamics [12]. Observations on the trends of mortality behind these fluctuations indicate that the rate of mortality change slows down and diverges from the exponential law at very old ages. Data on late-life mortality slow-down are controversial and fall into three groups: (a) an increase in late-life mortality at a slower rate than its exponential increase during the adulthood period, called deceleration [13–15], (b) a levelling-off, commonly called the mortality plateau, which is the saturation of mortality trajectory on a horizontal line corresponding to constant mortality rate [16–19] or (c) a decline of mortality with increasing age [10, 20]. The late-life mortality slow-down was observed for human [4, 21] as well as non-human populations [16, 22–24].

Theoretical studies of mortality dynamics are mainly devoted to two main problems, namely how the biological processes underlying mortality result into the exponential law and what are the causes of deviations from the exponential law that are observed at young and extremely old ages. Mathematical verifications of the exponential law have been performed from different points of view ranging from a genetic theory of population ageing [25] to the application of reliability theory to ageing and longevity [9].

The evolutionary theories that were proposed to explain ageing, and to answer the question of why organisms grow old and die, are mostly based on the assumption of a loss of selective significance of phenotypes developing during post-reproductive ages [26–28]. One such theory is introduced by Medawar and known as the mutation accumulation theory [29, 30]. Medawar's hypothesis states that gene alleles or mutations that are neutral at early life but deleterious at later life, escape natural selection and are transferred to the next generation before their deleterious effects become evident. Such mutations can therefore accumulate in the population by a genetic drift and reveal themselves via the diseases associated with the post-reproductive period. Another evolutionary theory, known as antagonistic pleiotropy, states that genes, with beneficial effects early in life but with deleterious actions later in life, could be favoured by selection and accumulate in the population [31]. The theory of disposable soma, proposed by Kirkwood [32, 33], postulates that organisms have a limited amount of energy and that specific gene mutations save energy for reproductive aspects by reducing the amount of energy used for maintenance, leading to non-reproductive damages. Ageing is therefore a result of the accumulation of damages that are not repaired by the organism [34]. These hypotheses take for granted the fact that the length of the reproductive period itself may depend on a number of genetic determinants associated with environmental and population factors.

There are also other attempts to explain the Gompertz law which are not based on evolutionary theories. One of such theories relates mortality to inadequate responses of the organism to energy demands and shows that the exponential increase of mortality is associated with a linear decrease of vitality (where vitality was defined as the capacity of an individual to resist damage) [35]. Sacher and Trucco have related the Gompertz law to the effect of stochastic and homeostatic forces [36, 37], and Shklovskii - to the exponentially rare escape of abnormal cells from immunological response [38]. Furthermore, Gavrilov and Gavrilova have applied the reliability theory to explain ageing and the Gompertz law by considering age-related failure kinetics of systems (machines) and their components [9]. They have shown that the rate of machines' failure as a function of age can reproduce the known mortality laws (Gompertz law, compensation effect, late-life deceleration) and therefore the reliability theory can be used to explain biological ageing.

The above mentioned compensation effect is another universal law of mortality (in addition to the Gompertz law) and refers to the inverse relationship (negative correlation) between the scale and shape parameters that characterise the Gompertz law. The compensation law states that if different populations of the same species demonstrate different mortality dynamics, then a high initial mortality rate (scale parameter) in a population is compensated with a low rate of change of mortality with age (shape parameter) or similarly a low initial mortality rate is compensated with a high rate of change of mortality with age [9, 35, 39].

Many mathematical models have been introduced to reproduce the observed mortality patterns [19, 40–43]. Some of them are designed to generate mortality patterns over the entire lifespan while others aim to reproduce a specific part of those patterns. For example, a function that outlines an inverse relationship between mortality and age, is used to generate the decline of mortality at very young ages [44] whereas the logistic-type and quadratic functions are used to create the late-life mortality plateau and the late-life mortality decline, respectively [9–11].

A number of studies consider the heterogeneity of populations to model and analyse their impact on the mortality dynamics [45–47]. The heterogeneity can be introduced in different ways. For example it can be based on an assumption that the population is comprised of cohorts (subpopulations) such that the members of each cohort, at a given age, face the same probability of death [12, 48]. For the reason that all the individuals of each cohort are exposed to identical mortality dynamics, each subpopulation is considered as homogeneous. However, in reality, each single individual has specific genetic, biological and physiological characteristics which contribute differently to a lifespan of each individual. Therefore, an alternative way to introduce the heterogeneity is based on the consideration that each single individual in a population has his own specific traits and faces certain mortality dynamics. The force of mortality acting upon each individual has then a cumulative effect on the mortality process in the entire population. In this approach, the heterogeneity at individual level can be represented in two different ways. The first one refers to individual frailty as to a measure of the chances of survival [46, 49]. In the second, an individual vitality process is used to reproduce human mortality patterns [50–53], and thus it focuses on the process leading up to death. Under this consideration, vitality is defined as a measure of survival capacity which declines over age and is subject to stochastic changes. The intrinsic mortality is then a result of complete loss of vitality (each individual is born with an initial level of vitality and dies when his vitality declines to zero), while extrinsic mortality occurs when an environmental challenge exceeds the (non-zero) vitality level. The model of vitality processes was used to analyse the time-evolution of intrinsic and extrinsic mortality in terms of changes in the parameters by fitting the model to consecutive period data [50, 52] and also to explore the late-life mortality plateaus [53]. Thus, the concept of heterogeneity is used, in all these works, to explain the deviations of mortality from the exponential growth at young and very old ages [54–57].

Recently another mathematical model of heterogeneous population was proposed and used for the analysis of human mortality dynamics across the entire lifespan [12] as well as for the analysis of the evolution of mortality dynamics in Sweden over the 20^th^ century [58]. The main feature of this model (distinguishing it from other existing models of heterogeneous population), is the assumption that the mortality rate in each subpopulation increases exponentially over all ages in a similar way to what is described by the Gompertz law. This assumption allowed for an accurate reproduction of the entire mortality pattern and an explanation of certain features of mortality dynamics and their evolution over time. It has been shown that the best fit to actual human mortality data is generally achieved by a four-subpopulation model and that the deviations of mortality from the exponential increase can be explained by such heterogeneity [12]. Furthermore, by fitting the four-subpopulation model to actual (Swedish) mortality data for consecutive periods over the 20^th^ century, it was shown that the population tends to become more homogeneous over time [58]. This analysis has also validated the applicability of the compensation effect of mortality to each subpopulation in the model.

Since the above model of a heterogeneous population has been shown to be extremely useful for analysis of mortality data, there arises a question of whether this model reflects the real structure of the population and, if yes, what quality underlies the heterogeneity? The present work is devoted to the above question and represents a natural extension of the previous studies [12, 58] based on this model. We start by comparing the considered model [12] with a wide-range of other commonly used parametric models and show that it is one of the best models when the judgement is made on the basis of the quality of fit to the observed data. We then show that the model can explain the apparent controversial observations for old-age mortality (deceleration, mortality plateau and decline) which are not in contradiction with one another, but reflect a similar and coherent process underlined by the heterogeneity of populations. Finally, we tackle the problem of the nature of a population's heterogeneity. Although the heterogeneity of populations can be conditioned by various factors such as disparities in life-style, environmental and socio-economic conditions, we analyse the case of genotypic difference between subpopulations. We presume that the responses to environmental factors are largely shaped by an organism's genetic landscape and certain gene polymorphisms can affect the dynamics of ageing and mortality. In this work we check whether the population dynamics of putative gene variations can be aligned with mortality dynamics of suggested distinct subpopulations. We assume that individuals belonging to different subpopulations differ by genotype and have differential resistance to environmental perturbations. Changes in the environment would favour different subpopulations in different contexts and their resultant differential mortality may have an impact on the dynamics of the mortality characteristic for a population. Furthermore, it was previously shown [58] that the evolution of mortality dynamics in Sweden over the 20^th^ century was for two reasons: changes in mortality dynamics of subpopulations, and changes in the structure of populations as represented by the fractions made by subpopulations (resulting to homogenisation of the population). While changes in the mortality dynamics of subpopulations are most likely driven by environmental changes [59, 60], the change in the population structure can be explained in terms of population dynamics. Based on the difference in mortality dynamics of subpopulations (and assuming that the difference is due to a single gene) we have calculated their relative Darwinian fitnesses and confirmed that the calculated fitnesses allow for an explanation and accurate reproduction of the homogenisation process of populations.

The paper is structured as follows. In the next section we introduce different mathematical models for mortality and describe the technique used for an evaluation of allele frequency dynamics in population genomics. We then show that the model of a heterogeneous population is one of the best models for fitting actual cohort mortality data over the entire lifespan (Subsection 1 in Results) or for ages over 80 (Subsection 2 in Results). We emphasise that it can explain controversial data on late-life mortality (Subsection 3 in Results). Finally, we show (Subsection 4 in Results) that the homogenisation of the heterogeneous population as revealed by the evolution of period mortality data in Sweden over the 20^th^ century can be explained by changes in allele frequency due to different fitnesses corresponding to different subpopulations. We conclude with a discussion of the obtained results and provide further arguments on genotypic differences between subpopulations having different mortality dynamics in the Discussion Section.

## METHODS

In this section we give a description of a few popular models which are commonly used for fitting mortality data (these models later will be compared with each other in terms of their fit to a given set of data). For the scope of this analysis, we use the so-called “parametric” models that are models expressing mortality rates across the lifespan with fixed (i.e. time-independent) parameters. We exclude therefore the models that also consider the time dependency of mortality patterns such as the notable Lee-Carter model. The description of parametric models is followed by a description of the method of calculating the selection process in a population of diploid organisms which is later used for the analysis of the evolution of heterogeneous populations.

### 1. Models of mortality

#### Exponential functions: Gompertz, Makeham and Weibull

The first developed parametric model and the one that remains the most notable in the literature is the Gompertz model [19]. The Gompertz model (or Gompertz law) states the exponential increase of mortality with age in a significant portion of lifespan (from sexual maturity to extremely old ages). According to the Gompertz law, the central death rate at age *x*, is given by

(1) $$m_{x}=\alpha e^{\beta x}$$

where α is the initial mortality rate (scale parameter) and β is the rate of change of mortality with age (shape parameter). It is remarkable that the Gompertz law does not only hold for human populations but also for other biological species [39].

The Makeham model [40] is an extension of the Gompertz law which represents the death rate as the sum of an age-dependent component (the Gompertz function) describing deaths due to age-related diseases or disorders, and an age-independent component (a constant γ) describing deaths due to external factors such as accidents or certain infectious diseases:

(2) $$m_{x}=\gamma +\alpha e^{\beta x}.$$

A third exponential parametric model is the Weibull model [61] which expresses the mortality rate as a power function of age:

(3) $$m_{x}=\alpha x^{\beta }.$$

According to these three exponential models, mortality rates diverge to infinity as age tends to infinity. The difference in concavity or convexity of these functions, and the difference in their initial values when x=0 ($m_{0}=\alpha$ for Gompertz, $m_{0}=\gamma +\alpha$ for Makeham and $m_{0}=0$ for Weibull), distinguish them in terms of their usage. The Gompertz and Makeham models are generally used to describe the mortality of biological species while the Weibull function is widely used to describe the ageing and failure rate of technical systems and devices [62, 63].

#### Logistic functions: Perks, Beard and Kannisto

The logistic-type functions which shape sigmoid curves are commonly used in the analysis of mortality dynamics at old ages. These curves saturate, reaching horizontal asymptote, and can therefore produce the late-life mortality plateau [64, 65]. The general form of a logistic curve is expressed as a four-parameter function:

(4) $$m_{x}=\gamma +\frac{\alpha e^{\beta x}}{1+\delta e^{\beta x}}$$

which is known as the Perks model.

Different variations of logistic function can be used in order to reduce the number of parameters. A three-parameter logistic function is formed by setting δ=α in equation (4) or the three-parameter function introduced by Beard [66] by setting γ=0. Also, a simple two-parameter logistic function used by Kannisto [65] is formed by setting γ=0 and δ=α in equation (4).

The logistic function in equation (4) saturates asymptotically to γ+α/δ as age increases while the Beard function tends to the constant α/δ. The Kannisto model has an asymptote equal to one and this model is used in a common procedure for the construction of life tables in order to smooth the noisy death rates observed at ages 80 and above [15].

The Gompertz and Makeham models could be considered as special cases of equation (4). If δ=0, equation (4) is transformed into the Makeham model and if γ=δ=0 – into the Gompertz law. However, in both these models, the mortality rate tends to infinity as age increases which is in contrast to the logistic-type functions and due to the elimination of the denominator from the logistic form.

#### Michaelis-Menten kinetics

Michaelis-Menten kinetics is an outcome of a well-known model in biochemistry that describes the dynamics of catalysed reactions [67]. The kinetics is represented by an equation which describes the saturation of a reaction rate when the substrate concentration is increasing. The Michaelis-Menten equation has also been used to model several other processes. For example, Monod who was working in the field of environmental engineering used this equation to model the growth rate of microorganisms as a function of the nutrient's concentration [68]. In this study, we suggest using the Michaelis-Menten equation (disregarding its parameters and variable terminology) to fit mortality data and to be compared with other asymptotic mortality functions that reproduce the mortality levelling-off at very old ages (i.e. the logistic-type functions). Following the form of the Michelis-Menten equation, the mortality at age x can be expressed as:

(5) $$m_{x}=\alpha \exp\left(\frac{\beta x}{1+\gamma x}\right)$$

#### Exponential-Quadratic function

An exponential-quadratic function (known also as the Coale-Kisker model) is usually used to fit mortality data and show the deceleration of mortality rate and its decline at very old ages [69]. The exponential-quadratic function is given by

(6) $$\ln\left(m_{x}\right)=\alpha +\beta x+\gamma x^{2},$$

where for a concave down parabola with a maximum point, γ should be less than zero.

#### Heligman-Pollard model

Heligman-Pollard model [43] is an eight-parameter function that can reproduce mortality patterns of the entire lifespan with sufficient accuracy. The model was originally formulated for the ratio of death and survival probabilities ($q_{x}/p_{x}$) and composed of three terms where the first term reflects the sharp decline of mortality at childhood, the second reflects the accidental hump that is observed during the reproductive period (ages 15-40), and the third term (which is a Gompertz function) reflects the exponential increase of mortality at post-reproductive ages:

(7) $$\frac{q_{x}}{p_{x}}=A^{{\left(x+B\right)}^{C}}+De^{-E{\left(\log(x)-log(F)\right)}^{2}}+GH^{x}.$$

The last term of the Heligman-Pollard model is usually modified to the logistic form $GH^{x}/(1+GH^{x})$ to allow the saturation of mortality at extremely old ages. In this study, we used the Heligman-Pollard model to fit actual mortality rates (which are best approximated by the central death rate, $m_{x}$) instead of the ratio $q_{x}/p_{x}$.

#### Model of heterogeneous population

Mathematically, the model of a heterogeneous population, which postulates the exponential mortality dynamics for constituent subpopulations, expresses the mortality rate $m_{x}$ at age x, as a sum of weighted exponential terms:

(8) $$m_{x}=\sum_{j=1}^{n}\rho_{j,x}\alpha_{j}e^{\beta_{j}x}=\sum_{j=1}^{n}\rho_{j,x}m_{j,0}e^{\beta_{j}x}$$

where the sub-index j indicates the j*-th* out of n subpopulation, $m_{j,0}$ is the central death rate at age *0* of subpopulation j, $\alpha_{j}$ is the initial mortality rate of the j*-th* subpopulation, and $\beta_{j}$ is its mortality coefficient which gives the rate of change of mortality with age [12, 58]. The weights $\rho_{j,x}$ are fractions formed by each subpopulation j at age x in the entire population, and their sum is equal to unity at all ages. Finally, the mortality rate at age *0* of the subpopulation j is equal to $\alpha_{j}$ and thus we have the relationship $m_{j,0}=\alpha_{j}$, which leads to the last term in equation (8). For clarity and consistency purposes, in the remaining part of this study the first subpopulation represents the one with the highest initial mortality (j=1), the second is the one with the second highest initial mortality level (j=2), etc.

### 2. Model for dynamics of alleles based on diploid genetics

Natural selection is an evolutionary process taking place within a population and states that individuals with certain heritable traits have the ability to survive and reproduce offspring more often than individuals deficient in those traits. Since these traits are heritable, the proportion of individuals carrying genotypes that express these traits is gradually increasing over time. Hence, natural selection similarly to the other primary evolutionary forces (mutation, migration and genetic drift) causes changes in allele frequencies in a population. The ability of any individual to pass genes to the next generation is determined by fitness. The more likely an individual is to survive and live long enough to mate and reproduce, the higher their fitness is. A measure of fitness can be given by an average number of offspring that are born from parents of a given genotype [70]. Selection is therefore conditioned by the variation of fitness between different genotypes. A simple model of natural selection that counts the frequencies of alleles (and subsequently the number of individuals with specific genotypes) over discrete generations is described in this section.

A diploid gene with alleles A and B splits the population into three groups of individuals having three distinct genotypes: AA, AB or BB. The notations p and q are used to denote the frequencies of alleles A and B respectively and the notations P, Q and R are used to define the frequencies of genotypes AA, AB and BB, where p+q=1 and P+Q+R=1. After a single step of random mating the frequencies of the three genotypes are $P=p^{2}$, Q=2pq and $R=q^{2}$ satisfying the Hardy-Weinberg equilibrium [71]. Each allele frequency can also be expressed in terms of genotype frequencies. In other words the frequency of an allele is equal to the frequency of homozygote genotype formed by two duplicates of that allele plus half of the frequency of the heterozygote genotype, that is:

$$p=P+\frac{1}{2}Q\text{ and }q=R+\frac{1}{2}Q.$$

The absolute fitness of each genotype (denoted as $w_{AA}$, $w_{AB}$ and $w_{BB}$ accordingly) is considered here by the average number of offspring produced by the individuals who carry this genotype. Relative fitness, i.e. the fitness of one genotype relative to that of another, is given by the ratio of their absolute fitnesses. Since this study deals with human populations, certain assumptions, i.e. organisms are diploid, reproduction is sexual and mating is random, are assured. It is also assumed that neither mutations or gene flows take place, and that stochastic effects due to genetic drift are negligible (the population size is large enough). Based on these assumptions the following formulas for the change of allele frequencies from generation i to generation i+1 can be derived:

(9a) $$p^{i+1}=P^{i+1}+\frac{1}{2}Q^{i+1}=\frac{w_{AA}p^{2}+w_{AB}pq}{\bar{w}}$$

(9b) $$q^{i+1}=R^{i+1}+\frac{1}{2}Q^{i+1}=\frac{w_{BB}q^{2}+w_{AB}pq}{\bar{w}}$$

where the denominator in both fractions is the normalised factor $\bar{w}=w_{AA}p^{2}+2w_{AB}pq+w_{BB}q^{2}$ [71], representing the average number of children per individual in the population of interest. The changes in genotype frequencies between two subsequent generations are shown in Table 1.

**Table 1:** Recurrence relation of genotype frequencies between two consecutive generations in a diploid genetics model with random mating

Genotype	AA	AB	BB
Frequency of genotype at generation i	$P=p^{2}$	Q=2pq	$R=q^{2}$
Absolute fitness	$w_{AA}$	$w_{AB}$	$w_{BB}$
Frequency of genotype at generation i+1	${\left(\frac{w_{AA}p^{2}+w_{AB}pq}{\bar{w}}\right)}^{2}$	$2\left(\frac{w_{AA}p^{2}+w_{AB}pq}{\bar{w}}\right)\left(\frac{w_{BB}q^{2}+w_{AB}pq}{\bar{w}}\right)$	${\left(\frac{w_{BB}q^{2}+w_{AB}pq}{\bar{w}}\right)}^{2}$

## RESULTS

In this section, the mortality models described earlier are fitted to actual mortality data for the entire lifespan in Subsection 1 and for very old ages (above age 80) in Subsection 2, and comparisons between the fits of the models are performed. The data used in this study are death rates for Swedish, Norwegian and Japanese populations (both sexes combined) taken from the Human Mortality Database (http://www.mortality.org, assessed July 2016) [72]. Nonlinear least squares regression is used to fit the models to the data as provided by the tool Solver in Microsoft Excel [73, 74].

A justified version of the Bayesian Information Criterion (BIC) [75] is used to evaluate the goodness-of-fit of the models. The BIC is defined as

$$BIC=n_{d}\ln\left(\hat{\sigma_{e}^{2}}\right)+k\ln(n_{d}),$$

where $n_{d}$ is the number of data points, $\hat{\sigma_{e}^{2}}$ is the sum of squared residuals divided by the number of data points and k is the number of free parameters [76, 77]. In Subsection 3 the study focuses on mortality at very old ages and shows that the model of a heterogeneous population can reproduce and explain various old-age mortality observations, namely deceleration, plateau and decline of mortality rate. In Subsection 4 the evolution of mortality dynamics in heterogeneous populations and specifically the homogenisation of populations over time is derived from the changes in genotype frequencies in successive generations through the process of natural selection.

### 1. Comparing mortality models by fitting data over the entire lifespan

**Figure 1:**
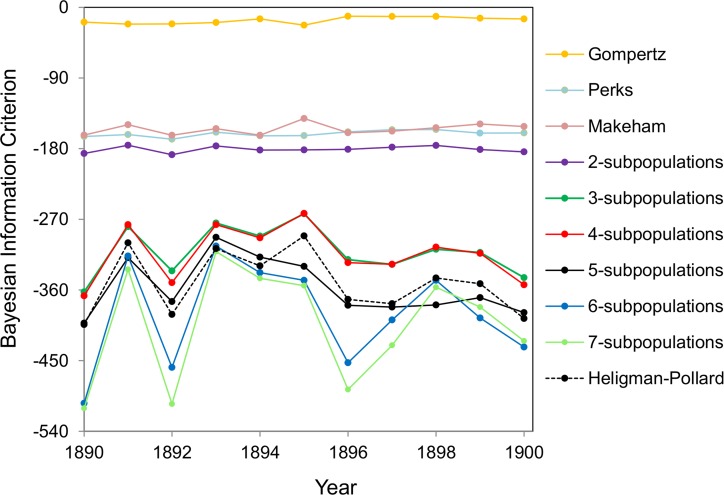
BIC values for different mortality models fitted to the Swedish 1890-1900 cohort mortality data The fits by Gompertz, Makeham, Perks and Heligman-Pollard models and the fits by the model of heterogeneous population consisting of two to seven subpopulations are shown.

**Figure 2:**
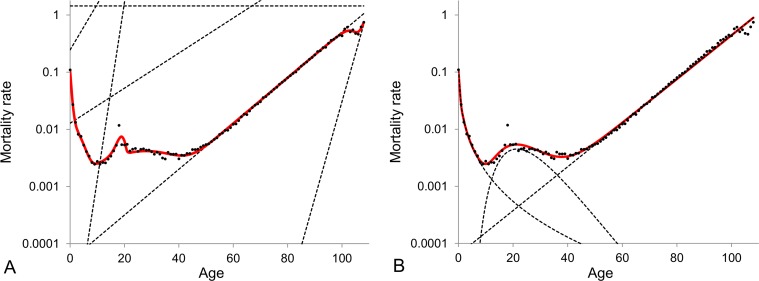
1900 cohort Swedish mortality data fitted by the model of heterogeneous population composed of six subpopulations (panel A) and the Heligman-Pollard model (panel B) The dots represent the observed central death rates, while the dashed curves in panel (**A**) indicate the exponential mortality dynamics of each subpopulation in the model of heterogeneous population and in panel (**B**) - the dynamics of the three components of the Heligman-Pollard model. Note that the plots are given in semi-logarithmic scale.

The model of heterogeneous population with different numbers of subpopulations as well as the Gompertz, Makeham, Perks and Heligman-Pollard models are fit- ted to cohort mortality data over the entire lifespan for the total (males and females) Swedish population. The BIC values as calculated by fitting the models to the 1890-1900 Swedish cohort data are shown in Figure 1. The model that gives the lowest BIC value provides the best fit to the data. The values in Figure 1 indicate that Gompertz, Makeham and Perks models are the weakest models in terms of data fitting. The model of heterogeneous population gets better with an increase in the number of subpopulations from two to six but any further increase in the number of subpopulations does not result in significant improvements. Also, the Heligman-Pollard model fits the data over the entire lifespan very well and is competitive to the model of heterogeneous population. Heligman-Pollard model fits data better than the model of heterogeneous population comprised of up to four subpopulations, but worse when the number of subpopulations increases to five or above. The actual fits of the six-subpopulation and Heligman-Pollard models to the 1900 cohort Swedish death rates are shown in Figure 2.

The Heligman-Pollard model fits the age-dependent mortality patterns very accurately since it imposes a pre-defined mortality pattern. Indeed the Heligman-Pollard model divides the mortality pattern into three distinct components observed over the past century, namely infant, accidental and adult mortality. On the other hand, the model of heterogeneous population is more abstract, since it does not impose any pre-specified pattern: it assumes that the most basic feature of biological populations are their heterogeneity and all peculiarities of population mortality dynamics are conditioned by interplay between mortalities of subpopulations. Subpopulations in turn are homo-geneous and their mortality dynamics simply follow the exponential law. The model can then be adapted to any dataset and can reproduce very different mortality curves. This flexibility allows (i) to fit mortality data very well for any part of the lifespan (see for example Subsection 2 on old ages), (ii) to reproduce different and potentially controversial observed mortality patterns (see Subsection 3 for an example related to old-age mortality) and (iii) to capture any new and thus un-expected mortality features (for example the reduction of external causes of death may result in the elimination of the accidental hump [78]).

### 2. Comparing mortality models by fitting data for ages beyond 80

In this section we focus on mortality at very old ages (above age 80) and analyse the phenomenon of late-life mortality divergence from the exponential dynamics. For this analysis we use the models designed for old age mortality described in Methods. All these models were fitted to cohort Swedish data for ages 80-109. We have fitted the models to single-year cohort data but the obtained results were not conclusive. Due to the scarcity of long-lived individuals, the statistical noise of mortality rates at old-ages is high and thus, it is difficult to draw any conclusions from single-year data as the optimal model differs across cohorts (i.e the Gompertz model, the logistic-type models or the heterogeneous population model reveal the best fit for some cohorts). Therefore, we decided to use more data in order to reduce the noise present in the data and thus, to be able to capture the underlying trend [79]. For model fitting we have used the data averaged for eleven birth cohorts (from 1890 cohort to 1900 cohort). The BIC values calculated by fitting the models to the data are shown in Table 2.

**Table 2:** Comparison of BIC values for several parametric models fitted to the averaged 1890-1900 Swedish cohort data for ages beyond 80

Model	Equation	Number of parameters	BIC	$\underset{x\to \infty }{\lim}m_{x}$
Gompertz	$m_{x}=\alpha e^{\beta x}$	2	−153.446	∞
Makeham	$m_{x}=\gamma +\alpha e^{\beta x}$	3	−150.045	∞
Weibull	$m_{x}=\alpha x^{\beta }$	2	−156.711	∞
Heterogeneous 2-subpopulations	$m_{x}=\overset{2}{\underset{j=1}{\sum^{\text{​}}}}\rho_{j,x}m_{j,0}e^{\beta_{j}x}$	5	−168.653	∞
Heterogeneous 3-subpopulations	$m_{x}=\overset{3}{\underset{j=1}{\sum }}\rho_{j,x}m_{j,0}e^{\beta_{j}x}$	8	−156.422	∞
Perks	$m_{x}=\gamma +\frac{\alpha e^{\beta x}}{1+\delta e^{\beta x}}$	4	−152.169	$\gamma +\frac{\alpha }{\delta }$
3-parameter Logistic	$m_{x}=\gamma +\frac{\alpha e^{\beta x}}{1+\alpha e^{\beta x}}$	3	−143.727	γ+1
Beard	$m_{x}=\frac{\alpha e^{\beta x}}{1+\delta e^{\beta x}}$	3	−155.570	$\frac{\alpha }{\delta }$
Kannisto	$m_{x}=\frac{\alpha e^{\beta x}}{1+\alpha e^{\beta x}}$	2	−134.444	1
Michaelis-Menten	$m_{x}=\alpha e^{\beta x/(1+\gamma x)}$	3	−156.843	$\alpha e^{\beta /\gamma }$
Exponential-Quadratic	$m_{x}=e^{\alpha +\beta x+\gamma x^{2}}$	3	−156.668	0 (for γ<0)

All model parameters are assumed to be greater than or equal to zero except for the parameters α and γ in the exponential-quadratic model where they are assumed to be negative.

The logistic-type models (Perks, 3-parameter logistic, Beard and Kannisto) and the Michaelis-Menten-type model show convergence to a certain limit as age increases and are therefore suitable to explain the late-life mortality deceleration and the existence of mortality plateaus. The exponential-quadratic model can generate a concave down parabola and therefore explains the decline of mortality at old ages. The exponential models by Gompertz, Makeham and Weibull fail to explain the late-life mortality slow down, because the death rates expressed by these functions tend to infinity as age increases. Even if the subpopulation mortality rates also diverge as age tends to infinity, the model of hetero-geneous population appears to be the only model which, due to interplay between subpopulations, can reproduce all observations (deceleration, plateau and decline) in mortality in later life.

**Figure 3:**
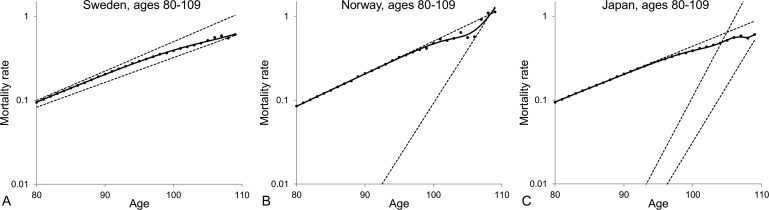
Model of heterogeneous population fitted to averaged 1890-1900 cohort death rates for ages over 80 for Swedish (A), Norwegian (B) and Japanese (C) populations The dots represent the observed central death rates, while the exponential mortality dynamics of the subpopulations are shown by the dashed lines and the mortality dynamics of the entire population are shown by the black solid lines. Note that the plots are shown on a semi-logarithmic scale.

**Figure 4:**
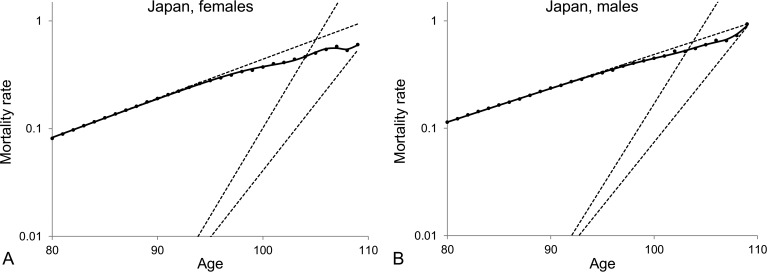
Model of heterogeneous population composed of three subpopulations fitted to averaged 1890-1900 cohort death rates for ages over 80 for Japanese female (A) and Japanese male (B) populations The dots represent the observed central death rates, while the exponential mortality dynamics of the subpopulations are shown by the dashed lines and the mortality dynamics of the entire population are shown by the black solid lines. Note that the plots are shown on a semi-logarithmic scale.

From the results shown in Table 2, one can conclude that the model of a heterogeneous population composed of two subpopulations provides the best fit to the mortality data at old ages. For the averaged Swedish data, the mortality curve generated by the model of a heterogeneous population increases exponentially, asymptotically to the level of the dynamics of the frailest subpopulation between ages 80 and 90, then decelerates to reach the level of the dynamics of the most robust subpopulation and then keeps increasing exponentially at that level (Figure 3A). Similar results and conclusions have been derived by fitting the models presented in Table 2 to the death rates of ages 80+ for other developed countries, including Norway (Figure 3B) and Japan (Figure 3C). Interestingly, the Japanese data are better fitted by the three-subpopulation model. The trajectory of mortality that fits the Japanese data increases exponentially along the level of the frailest subpopulation then decelerates for a couple of years, then creates a plateau and finally re-accelerates after the age of 108. Besides, similar results are obtained when males and females are analysed separately, as illustrated on Figure 4 with Japanese mortality data.

On the basis of the analysis presented in Figures 3 and 4 we conclude that different observations on mortality dynamics at extremely old ages can be explained by the heterogeneity of populations, which is further developed in the following section.

### 3. Late-life mortality slow-down due to population heterogeneity

**Figure 5:**
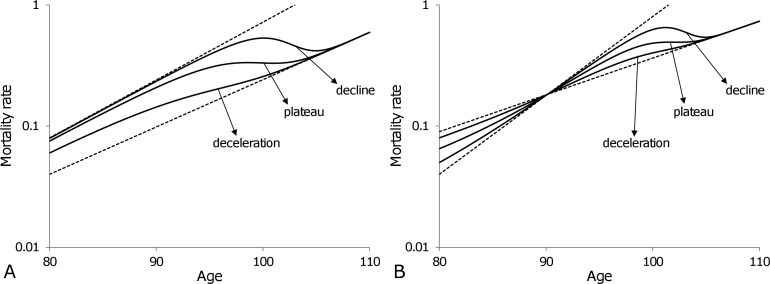
Theoretical trajectories (solid curves) of old-age (80-110) mortality dynamics for a heterogeneous population composed of two subpopulations Variations in relative sizes of the subpopulations permit the reproduction of all three observations for late-life mortality: deceleration, plateau and decline. Once the individuals of the frailest subpopulation die out, the mortality of the entire population follows the exponential dynamics of the most robust subpopulation. In panel (**A**) the same subpopulation remains frailest over all ages, while in panel (**B**) the subpopulation which is frailest before age 90 becomes the most robust after age 90. Note that the plots are shown on a semi-logarithmic scale.

Heterogeneity suggests that late-life mortality slow-down is a result of the variation in robustness between sub-cohorts having a significant number of survivors at old ages. In addition, heterogeneity permits us to explicate three different observations in late-life mortality, namely deceleration, saturation and the decline of mortality rates. Figure 5 shows that the simple model of a heterogeneous population composed of only two subpopulations can reproduce all these observations. In Figure 5A the frailest subpopulation (i.e. the subpopulation that dies out fastest) is the one that has the highest mortality rate at age 80, $m_{1,80}=0.08$, and the highest mortality coefficient, $\beta_{1}=0.11$, as compared to the most robust subpopulation that has a mortality at age 80 of $m_{2,80}=0.04$, and a mortality coefficient of $\beta_{2}=0.09$. The variation in the proportions of the two subpopulations determines the formation of the three different late-life phenomena. For example, if the fraction of the frailest subpopulation at age 80 in Figure 5A is $\rho_{1,80}=0.5$, then the overall mortality of population shows a deceleration, if $\rho_{1,80}=0.88$ - a plateau and if $\rho_{1,80}=0.98$ - a decline.

A mortality cross-section, shown in Figure 5B, occurs when one of the subpopulations has a lower mortality rate than the other at younger ages, but higher at older ages (i.e. it is more robust initially but becomes frailer after a cross-section). In particular, the theoretical subpopulations presented in Figure 5B have mortality rates at age 80 and mortality coefficients $m_{1,80}=0.09$, $\beta_{1}=0.07$ and $m_{2,80}=0.04$, $\beta_{2}=0.15$ respectively. The fractions $\rho_{2,80}=0.2,\;0.5$ and 0.8 for the subpopulation with the lowest mortality rate at age 80 are used to reproduce deceleration, plateau and decline respectively.

The mortality trajectories presented in Figure 5 illustrate that apparently controversial observations in mortality dynamics for old ages are not necessarily in contradiction with each other and can be explained by the heterogeneity of populations.

### 4. Evolution of mortality dynamics: homogenisation and natural selection

The model of a heterogeneous population [12] have previously been used to analyse the evolution of Swedish period death rates over the 20^th^ century [58]. In a view of incompleteness of cohort data for this century the analysis was done on the basis of period data. Although the model of heterogeneous population is designed primary for dealing with cohort data it was found that on the basis of period data one can still make reliable conclusions. It was shown that the best fit model involves four subpopulations: the first subpopulation reproduces the initial decline of mortality for infants, the second - the mortality at childhood, the third - the accidental mortality during reproductive period and the fourth - the exponential (Gompertz) growth of mortality at adult span. The analysis of mortality evolution, as examined by using this model, showed that the parameters which characterise the mortality dynamics of each subpopulation evolve through time displaying two remarkable features. The first is the confirmation of the compensation effect for each evolving subpopulation, and the second is the homogenisation of the entire population manifested by the reduction in the initial fractions of the first three subpopulations (that are also the smallest subpopulations) and an increase in the initial fraction of the fourth subpopulation (from 67% at the beginning of the 20^th^ century to 99% at its end).

An alternative way to examine the evolution of Swedish mortality dynamics over the 20^th^ century is to modify the model of heterogeneous population by making parameters time-dependent and fitting the model to the entire set of period mortality data over age and time so that the fit will be represented on a three-dimensional surface. The death rates for ages 0 to 100 and for the one-century period (101 years from 1900 to 2000) compose a dataset of 10201 points. On the other hand, the four-subpopulation model has 12 parameters of which 11 are independent (the condition that the sum of the fractions $\rho_{j,x}$ at each age is equal to unity reduces by one the number of free parameters). Each of the 11 parameters is assumed to change linearly or exponentially over time according to their trend-lines [58]. Each linear or exponential trend is characterised by two parameters (a scale and a shape parameter) and therefore the modified time-dependent model has 22 free parameters. Thus, this approach requires the estimation of the values of only 22 parameters in order to fit the 10201 data points while in order to fit data separately for each year [58] one would require to estimate the values of 11 unknown parameters for each of 101 data points (or in other words, 1111 unknown parameters in total to fit the 10201 data points).

**Figure 6:**
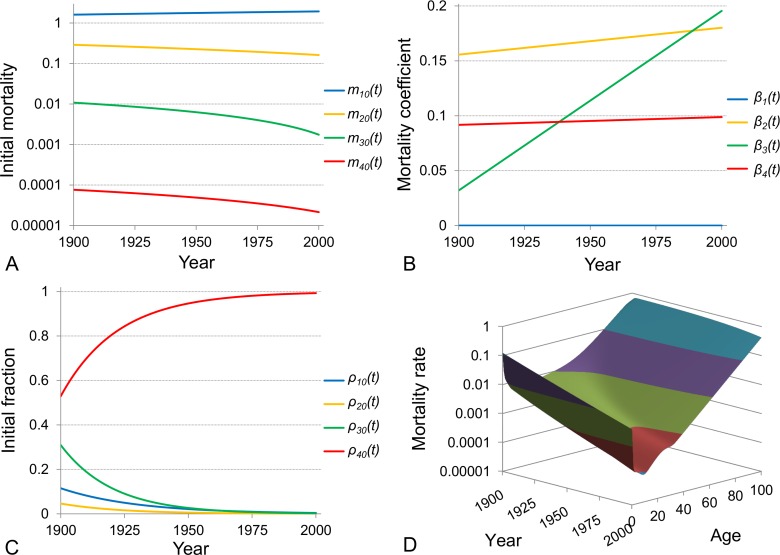
Time-evolution of mortality dynamics in the mathematical model of a heterogeneous population The model of a heterogeneous population composed of four subpopulations is modified to contain time-dependent parameters and is used to fit period Swedish death rates for ages 0 to 100 and for the entire 20^th^ century period (1900-2000). The resulting fitted surface of the modified model to the age- and time-related Swedish data is shown in panel (**D**). The initial mortalities and the mortality coefficients of subpopulations are assumed to change linearly over time (fits are shown in panels (**A**) and (**B**) respectively) while their initial fractions change exponentially (shown in panel (**C**)). Note that the plot in panel A is shown on a semi-logarithmic scale.

The 3-dimensional surface that is reproduced by fitting the modified model to age- and time-related Swedish data is shown in Figure 6D. The initial mortalities $m_{j,0}$ and the mortality coefficients $\beta_{j}$ for each subpopulation j are assumed to change linearly over time as shown in Figures 6A and 6B respectively. The negative correlation between the initial mortality and the mortality coefficient in each subpopulation indicates the validation of the compensation law of mortality. The initial fractions of the four subpopulations are assumed to change exponentially over time (Figure 6C). The phenomenon of homogenisation is evident as the initial fraction of the most robust subpopulation (red line in Figure 6C) increases over time and dominates at the end of the century, while the fractions of the other three sub-populations decrease and these subpopulations almost disappear by the end of the century. The most robust subpopulation has the smallest initial mortality rate, and more individuals belonging to this subpopulation survive to more advanced ages compared to the individuals from the other subpopulations.

Further examination of the results shown in Figure 6 indicates that all individuals from the first two subpopulations, reflecting infant and child mortality, die before sexual maturity and the reproductive period and therefore they do not leave offspring. The other two subpopulations have individuals that survive till reproductive age and consequently leave offspring who contribute to the next generation. However, the most robust subpopulation contributes relatively more and if we assume that these two subpopulations differ by genotype, the evolution of their initial fractions can be explained by natural selection. This problem is addressed in the following part of our study, namely we assume that the third and fourth subpopulations differ by a single gene (which has two alleles) and check whether the change in the fractions of these subpopulations follows the changes in allele frequencies over generations due to natural selection.

We use the model for evolution of allele frequencies in diploid organisms as described in Methods and assume that alleles A and B indicate two distinct traits related to mortality dynamics. Choosing from two possibilities we pick up on an assumption that the allele A is dominant and therefore the heterozygotes AB have the same mortality-related phenotype as the homozygotes AA. Furthermore, the individuals carrying genotypes AA and AB are assumed to belong to the third sub-population while individuals with BB genotype belong to the fourth. To calculate Darwinian fitness, we make the following simple assumptions concerning the reproductive behaviour of the individuals who make up the population: (1) reproductive behaviour does not depend on genotype (note that mortality depends on genotype and makes the fitness genotype specific); (2) reproductive age is set from the age of 20 to the age of 40; (3) within this age interval, reproduction takes place with the constant probability, ϕ, at any age (i.e. it is the same for both subpopulations and independent of age). We believe that by using these assumptions we can obtain a relatively good approximation of the spreading process of a favourite allele in the population due to its effect on mortality only, and thus the dynamics of the relative sizes of two subpopulations. For a more precise analysis, one can adjust the model assumptions by taking into account real fertility related data, the age dependence of reproduction probability, and by specifying the reproductive age-interval more accurately (which is different for males and females). However, here we keep the model as simple as possible and leave various extensions to the framework, which we are introducing here, for future studies.

Based on the above assumptions we can calculate the absolute fitnesses of individuals that belong to the third and the fourth subpopulations which will be denoted by $w_{3}\;(=w_{AA}=w_{AB})$ and $w_{4}\;(=w_{BB})$ respectively. Fitnesses can be evaluated based on mortality dynamics and expressed as functions of the parameters that describe the exponential mortality dynamics of these two subpopulations as shown in Table 3. In this table, $N_{j,0}$ represents the number of individuals in subpopulation j at age 0.

**Table 3:** Genotype frequencies and fitnesses in terms of the model parameters

Subpopulation	3^rd^	4^th^
Genotypes	AA+AB	BB
Initial fraction	$\rho_{3,0}=P+Q=p^{2}+2pq$	$\rho_{4,0}=R=q^{2}$
Absolute fitness	$w_{3}=\phi \overset{40}{\underset{x=20}{\sum }}N_{3,0}\exp\left(\frac{m_{3,0}}{\beta_{3}}\left(1-e^{\beta_{3}x}\right)\right)$	$w_{4}=\phi \overset{40}{\underset{x=20}{\sum }}N_{4,0}\exp\left(\frac{m_{4,0}}{\beta_{4}}\left(1-e^{\beta_{4}x}\right)\right)$
Relative fitness	$w_{3}/w_{4}$	1

The absolute fitness of individuals belonging to each subpopulation is calculated as a total number of person-years within the reproductive period (assumed to cover ages 20 to 40) multiplied by a probability to reproduce, ϕ, which is assumed to be age-independent and constant for all individuals. Note that since the initial mortality and mortality coefficient of each subpopulation change over time, the absolute and relative fitnesses also change over time.

The absolute and relative fitnesses of subpopulations are found using their initial mortalities and mortality coefficients which are obtained by fitting the heterogeneous model with four subpopulations to the Swedish period data. The estimated initial fractions of two subpopulations (third and fourth), which are involved in reproduction, for the period 1900 (starting point of the examined time-interval) are normalised to have a sum equal to one (since we do not consider subpopulations 1 and 2 which are not involved in reproduction) and are then used to calculate the frequencies of alleles A and B (or values of p and q) in 1900.

**Figure 7:**
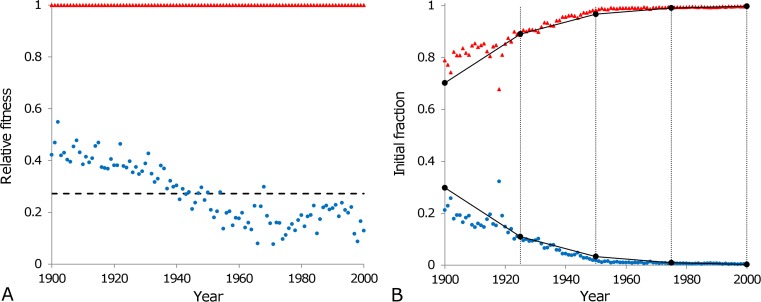
Population homogenisation as a consequence of natural selection The relative fitnesses of individuals belonging to the third (blue circles) and the fourth (red triangles) subpopulations, as calculated according to the formulas in Table 3, are shown in panel (**A**). The relative fitness of the third subpopulation varies from year to year with an average value of 0.27 (black dashed line) over the entire century. This average value is used to calculate the changes in genotype frequencies due to natural selection as shown in panel (**B**) (black circles), over four generations (each lasting 25 calendar years as indicated by the vertical lines in panel (**B**). Calculated genotype frequencies are interpolated linearly (solid lines connecting circles in panel (**B**) within each generation to be comparable with the normalised initial fractions of the third (blue circles) and the fourth (red triangles) subpopulations modeled to fit the Swedish data for the period 1900 to 2000.

Possessing all of the above considerations and the equations that describe the flow of alleles due to selection (equations 9a and 9b), the changes of genotype frequencies over generations are calculated presuming that each generation corresponds to 25 calendar years totalling four generations per century. Following this, changes in genotype frequencies are compared with the evolution of the initial fractions in the model of heterogeneous population over the 20^th^ century. The outcome of this analysis is shown in Figure 7. Thus, assuming that the difference in mortality dynamics of two subpopulations is conditioned by a difference in a single gene and taking an average value of relative fitness (black dashed line in Figure 7A), we calculate how the relative fractions of the subpopulations (corresponding to AA+AB genotype frequency for subpopulation 3 and BB genotype frequency for subpopulation 4, black lines in Figure 7B) evolve due to natural selection. We can state that the obtained result is surprisingly close to the changes of the fractions (red triangles and blue dots in Figure 7B) in the best-fit model of heterogeneous population. The significance of this result is that it serves as a self-consistency test for the model of heterogeneous population [58].

Our further calculations have shown that dividing the century into three or five generations (i.e. the time interval between generations is 33 or 20 years respectively) does not significantly change the genotype frequencies observed in Figure 7B (frequencies decrease/increase by about 15%). Averaging fitnesses of subpopulations over each generation rather than over the entire century, does not significantly change the obtained results either (note that in the model, where the evolving population is discretised into generations, consideration of fitnesses for each year or for any intervals shorter than the duration of the generation does not make sense).

## DISCUSSION

The aim of this study was to show the advantages of the model of heterogeneous population (introduced in [12]) in terms of fitting the mortality data and to give a biological justification supporting this model. This was done in a few steps. In the first step we compared this model with a number of other parametric models which were used to fit actual mortality data. We found (Subsections 1 and 2 in Results) that the model of heterogeneous population is advantageous compared to the other models as it has the flexibility to be adapted to any dataset and therefore provides the best fit to mortality data for the data over the entire lifespan as well as for old (over 80) ages. In the second step we demonstrated (Subsections 3 in Results) that contrary to other considered models, the model of heterogeneous population can reproduce and explain controversial observations in late-life mortality (deceleration, plateau and decline). In the third step we assumed that population heterogeneity reflects the genetic variation between subpopulations, and showed (Subsection 4 in Results) that the natural selection model based on differential mortality can explain and quantitatively reproduce the homogenisation of the Swedish population within a one-century period. Based on these results, we conclude that heterogeneity, beyond its convenient use in reproducing characteristics of age-structured populations, has a fundamentally inherent role in understanding the mortality dynamics across the lifespan and the evolution of these dynamics over time.

The model of Heligman-Pollard is known for its excellent fit to the mortality data over the entire lifespan. Here we have shown that the model of heterogeneous population can provide with even better fit to that data. Besides, it is important to note that these two models are very different in nature. While the Heligman-Pollard model imposes a pre-defined mortali-ty pattern, the model of heterogeneous population allows the mortality pattern to be adapted to the fitted data. Both models are thus extremely useful in different contexts. The Heligman-Pollard model is better for forecasting purposes, as it avoids projecting unrealistic patterns far into the future. However, as a wide variety of mortality patterns can be modeled using a different number of subpopulations, the model of heterogeneous population allows us to capture new and unexpected patterns, providing a greater flexibility in data modeling. In addition to this, in the model of hetero-geneous population, the model parameters do not lose their interpretation in demographic terms, even with an increase in the number of parameters in the model. This flexibility is important for data analysis as mortality patterns evolve through time due to several factors (medical improvements, changes in life-style conditions, biological evolution, etc.) and this can be modeled as some subpopulations die out and some new ones become more pronounced in a quantitative sense. Furthermore, mortality dynamics of subpopulations change over time and these changes can be detected via fitting procedures.

The population heterogeneity as defined in [12] is significantly different from the heterogeneity as defined in other models including those based on vitality processes [50–53]. According to [53] the heterogeneity results from two components, an evolving one and an initial one. A cohort is initially homogeneous if the individuals are assumed to have the same vitality at birth. In initially homogeneous cohort the heterogeneity emerges if the vitality of individuals in the cohort changes differently (i.e. has stochastic component). It is a form of evolving heterogeneity in survival capacity. In contrast, the initial heterogeneity refers to the hetero-geneity in survival capacity at birth. A distribution of the initial vitality is developed and thus the individuals of a cohort have different vitality level at birth. The heterogeneity in our model is rather similar to the initial heterogeneity, that is the subpopulation fractions in our model are the analogue to the initial vitality distribution in [53]. While the time-evolution of the evolving heterogeneity was discussed in [50, 52], the time-evolution of the initial heterogeneity has not been analysed yet. Therefore, the homogenization of the population that is discussed within our model framework cannot be directly compared to models based on vitality processes. However, [50] and [53] mention that the initial vitality can, in part, be determined by genetic inheritance, which is in line with the core assumption used in our paper in order to explain the homogenisation of the population.

The introduction of subpopulations with different mortality characteristics can easily be justified on the basis of biological and medical observations. Certain diseases tend to follow others due to strong associations at genetic and cellular levels, and connections at the cellular level get amplified at the population level when a number of diseases emerge as comorbid [80–82]. Susceptibility to a particular disease may stratify a part of population to having a particular dynamic of age-related accumulation of other associated diseases, and consequently a specific dynamic of mortality. To some extent this process is related to one described in the reliability theory [9], where age-related failure kinetics will be different for particular human physiological systems and their components, and will have a different impact on different human subpopulations. More studies are required on accumulation trends for particular diseases with an emphasis on mortality curves on one hand, and underlying genomic factors on the other. We believe that the complex structure of human populations in respect to evolving disease patterns in different age-groups will be revealed from these studies and more evidence will be available for refining the mathematical models. Having this knowledge we may be able to understand and better predict mortality dynamics in complex human populations by using a pallet of primary disease-associated genomic markers.

Our analysis of the evolution of allele frequencies (under the assumption that genomic differences are responsible for the difference in mortality rates between subpopulations) has indicated that the homogenisation of the Swedish population in the 20^th^ century can be explained by the selection process in favour of a particular subpopulation better fitted to a changing environment during the studied period. The force of selection as calculated on the basis of mortality-related heterogeneity of the population is known as the force of mortality selection [54]. To provide an intuitive explanation of this force we note that the individuals belonging to frail subpopulations tend to die at younger ages (and more frequently before the reproductive ages) than ones from more robust subpopulations. Therefore frail subpopulations leave less offspring than more robust subpopulations. Consequently, the proportion of individuals belonging to more robust subpopulations increases through generations. Although we do not aim to propose a fully-specified and completely realistic evolutionary model, we show that using very simple assumptions we can relate the evolution of the heterogeneous structure of populations to genetics and natural selection. Thus this paper paves the way for many potential extensions regarding genetics and evolutionary theories.

There are many studies indicating that the currently observed increase in longevity is primarily associated with environmental changes [83]. In order to link this statement with our results on the evolution of allele frequencies, we would suggest considering the following hypothetical scenario. Consider the population carrying a gene with two alleles A and B. There are three different kinds of individuals: AA, AB and BB in this population for which we can assume the same pattern of mortality and reproduction (three identical subpopulations) so that the genetic structure of this population is in equilibrium. Now assume that due to some environmental change the mortality of individuals carrying BB is reduced. If this reduction hits the reproductive period, the frequency of B will tend to increase which will result in a change to the structure of the population and to gradual change in its mortality dynamics causing an increase in longevity. If the initial frequency of allele A is small then a jump in mortality patterns in cohort data should be observed with no further evolution. Contrary to this, if the initial frequency of allele B is small, then the jump will be replaced by a gradual evolution, associated with an increase of allele B frequency. Obviously the period data should show a gradual evolution of mortality patterns in both cases.

In this paper we focus on two subpopulations whose mortality dynamics evolve differently (in response to the same environmental changes) and this shows a change in the overall mortality pattern. Mortality patterns of both subpopulations change over the 20th century but for our analysis we have averaged the characteristics of the subpopulations by taking their average fitness. Thus we have reduced our analysis to the following idealised case: environmental change has happened on or before 1900 and this has changed the mortality patterns for subpopulations 3 and 4. The latter causes the changes in fitnesses of subpopulations, follow up gradual changes in the population structure and consequently lead to gradual increase in longevity. We do not address the question of why mortality of subpopulations changes in a certain way (in response to environmental changes), but taking these changes as granted we confirm that the change in the structure of populations (represented by fractions of subpopulations) correlates with the evolution of frequency of the hypothetical allele.

The surprising part of this result is the time scale of the process: the selection process causes significant changes to take place in the population within one century (over four generations). In the model we have assumed that there is no difference in the reproductive behaviour of individuals belonging to different subpopulations and thus the difference in fitnesses is only conditioned by the difference in mortality patterns of the sub-populations, namely by their initial mortalities and mortality coefficients. As these parameters are time dependent, the relative fitnesses of subpopulations also change over time. In our study illustrated in Figure 7, we have ignored the fact that the fitnesses of the subpopulations evolve over time and used the average fitnesses over the entire century to calculate changes during four generations. This was done for two reasons: (1) to illustrate the process in a very simple case when the fitnesses do not change over time and (2) to properly account for variations in fitnesses from year to year, we would have to give up the idea of discrete generations and design a much more sophisticated model (i.e. design a virtual population).

An interesting question concerning the evolution of subpopulations analysed in Figure 7, concerns the relationship between them in the 19^th^ century. Our preliminary study shows that subpopulation 3 (which is almost extinct by the end of the 20^th^ century) had a higher relative fitness for most of the 19^th^ century as its mortality rate was lower than that of the fourth subpopulation and as a consequence the fraction of subpopulation 3 was increasing in the 19^th^ century (and then decreased in the 20^th^ century).

In this paper we have presented a very simple, almost caricature, natural selection model to compare its outcome with the evolution of subpopulations in the fits of heterogeneous model to mortality data. Surprisingly close correspondence between the time evolution of the subpopulation in the model of heterogeneous population and the evolution of genome frequencies can already be highlighted on the basis of this model. This finding naturally paves the way for many interesting research questions and future research studies associated with the development of more realistic models based on genetics and natural selection. More complex models should be based on more accurate representation of reproduction patterns and take into account the effect of more than one gene polymorphism and naturally occurring splits in frequencies of different gene variants.

Several additional extensions of this work are foreseen. The first one is associated with the impact of the environment on mortality changes. Indeed, the sharp reduction in overall mortality during the 20^th^ century and especially the dramatic decline of premature (infant and child) mortality in almost all countries is mainly a result of environmental changes and improvements [59, 60] and to a lesser extent, biological evolution. However, in our model framework, we do not explicitly account for environmental factors. Each subpopulation reacts in its own way to the environmental changes, and the mortality pattern of each subpopulation (here the scale and shape parameters of their exponential dynamics shown in Figures 6A and 6B) evolves differently over time. Therefore, we should further explore the effects of environmental change on mortality dynamics of heterogeneous populations (that would be reflected in the evolution of the model parameters), on reproductive success, reproduction windows and duration of lifespan (interesting results can be found in [84, 85]). Second, the consideration of the heritability of phenotypic mortality-related traits which are affected by genetic variations and environmental factors would be of great value. Third, future research could involve deeper consideration of the age-dependent fertility rate, male-female ratio, wider or narrower reproductive periods and changes over time including time-dependent fitnesses. Fourth, the effects of in and out migration, mutations and genetic drift could be examined. Finally an extensive literature exists on biological ageing and its potential relation to some longevity genes. Linking this stream of research with the model proposed in this study could allow development of new mortality modeling tools and lead to further accumulation of knowledge on mortality and longevity matters.

## References

[1] Carnes BA, Olshansky SJ, Grahn D (1996). Continuing the Search for a Law of Mortality. Popul Dev Rev.

[2] Jones HB (1956). A special consideration of the aging process, disease, and life expectancy. Adv Biol Med Phys.

[3] Finch CE (1994). Longevity, senescence, and the genome.

[4] Greenwood M, Irwin JO (1939). The biostatistics of senility. Hum Biol.

[5] Olshansky SJ, Wachter KW, Finch CE (1998). On the biodemography of aging: a review essay. Popul Dev Rev.

[6] Gavrilova NS, Gavrilov LA (2014). Mortality trajectories at extreme old ages: a comparative study of different data sources on U.S. old-age mortality. Living 100 Monogr.

[7] Gavrilov LA, Gavrilova NS (2011). Mortality Measurement at Advanced Ages: A Study of the Social Security Administration Death Master File. N Am Actuar J.

[8] Gavrilova NS, Gavrilov LA (2015). Biodemography of old-age mortality in humans and rodents. J Gerontol A Biol Sci Med Sci.

[9] Gavrilov LA, Gavrilova NS (2001). The reliability theory of aging and longevity. J Theor Biol.

[10] Kannisto V, Lauritsen J, Thatcher AR, Vaupel JW (1994). Reductions in mortality at advanced ages: several decades of evidence from 27 countries. Popul Dev Rev.

[11] Pham H (2011). Modeling U.S. mortality and risk-cost optimization on life expectancy. IEEE Trans Reliab.

[12] Avraam D, de Magalhaes JP, Vasiev B (2013). A mathematical model of mortality dynamics across the lifespan combining heterogeneity and stochastic effects. Exp Gerontol.

[13] Depoid F (1973). Mortality of old people over 85. Population (Paris).

[14] Horiuchi S, Wilmoth JR (1998). Deceleration in the age pattern of mortality at older ages. Demography.

[15] Thatcher AR, Kannisto V, Vaupel JW (1998). The force of mortality at ages 80 to 120.

[16] Economos AC (1979). A non-Gompertzian paradigm for mortality kinetics of metazoan animals and failure kinetics of manufactured products. Age (Omaha).

[17] Mueller LD, Rose MR (1996). Evolutionary theory predicts late-life mortality plateaus. Proc Natl Acad Sci USA.

[18] Curtsinger JW, Gavrilova NS, Gavrilov LA (2006). Biodemography of aging and age-specific mortality in Drosophila melanogaster. Handbook of the biology of aging.

[19] Gompertz B (1825). On the nature of the function expressive of the law of human mortality, and on a new mode of determining the value of life contingencies. Philos Trans R Soc Lond.

[20] Wilmoth JR (1995). Are mortality rates falling at extremely high ages? An investigation based on a model proposed by Coale and Kisker. Population Studies.

[21] Bebbington M, Green R, Lai C-D, Zitikis R (2014). Beyond the Gompertz law: exploring the late-life mortality deceleration phenomenon. Scand Actuar J.

[22] Carey JR, Liedo P, Orozco D, Vaupel JW (1992). Slowing of mortality rates at older ages in large medfly cohorts. Science.

[23] Pletcher SD, Curtsinger JW (1998). Mortality plateaus and the evolution of senescence: why are old-age mortality rates so low?. Evolution.

[24] Economos AC (1980). Kinetics of metazoan mortality. J Soc Biol Struct.

[25] Charlesworth B (1994). Evolution in age-structured populations.

[26] Hamilton WD (1966). The moulding of senescence by natural selection. J Theor Biol.

[27] Fisher Charlesworth B, Medawar (2000). Hamilton and the evolution of aging. Genetics.

[28] Rose MR, Rauser CL, Benford G, Matos M, Mueller LD (2007). Hamilton's forces of natural selection after forty years. Evolution.

[29] Medawar PB (1946). Old age and natural death. Modern Q.

[30] Medawar PB (1952). An unsolved problem of biology.

[31] Williams GC (1957). Pleiotropy, natural selection, and the evolution of senescence. Evolution.

[32] Kirkwood TB (1977). Evolution of ageing. Nature.

[33] Kirkwood TB, Holliday R (1979). The evolution of ageing and longevity.

[34] Kirkwood TB, Austad SN (2000). Why do we age?. Nature.

[35] Strehler BL, Mildvan AS (1960). General theory of mortality and aging. Science.

[36] Sacher GA, Trucco E (1962). The stochastic theory of mortality. Ann N Y Acad Sci.

[37] Yashin AI, Iachine IA, Begun AS (2000). Mortality modeling: a review. Math Popul Stud.

[38] Shklovskii BI (2005). A simple derivation of the Gompertz law for human mortality. Theory Biosci.

[39] Gavrilov LA, Gavrilova NS (1991). The biology of life span: a quantitative approach.

[40] Makeham WM (1860). On the law of mortality and the construction of annuity tables. The Assurance Magazine, and Journal of the Institute of Actuaries.

[41] Thiele PN (1872). On a mathematical formula to express the rate of mortality throughout the whole life. J Inst Actuar.

[42] Siler W (1979). A competing-risk model for animal mortality. Ecology.

[43] Heligman L, Pollard JH (1980). The age pattern of mortality. J Inst Actuar.

[44] De Beer J, Janssen F (2014). The NIDI mortality model. A new parametric model to describe the age pattern of mortality.

[45] Vaupel JW, Yashin AI (1985). The deviant dynamics of death in heterogeneous populations. Sociol Methodol.

[46] Vaupel JW, Manton KG, Stallard E (1979). The impact of heterogeneity in individual frailty on the dynamics of mortality. Demography.

[47] Manton KG, Stallard E, Vaupel JW (1986). Alternative models for the heterogeneity of mortality risks among the aged. J Am Stat Assoc.

[48] Vaupel JW, Yashin AI (1985). Heterogeneity's ruses: some surprising effects of selection on population dynamics. Am Stat.

[49] Yashin AI, Vaupel JW, Iachine IA (1994). A duality in aging: the equivalence of mortality models based on radically different concepts. Mech Ageing Dev.

[50] Anderson JJ, Li T (2015). A Two-Process Mortality Model with Extensions to Juvenile Mortality, Population Dynamics and Evolution.

[51] Anderson JJ, Gildea MC, Williams DW, Li T (2008). Linking growth, survival, and heterogeneity through vitality. Am Nat.

[52] Li T, Anderson J (2013). Shaping human mortality patterns through intrinsic and extrinsic vitality processes. Demogr Res.

[53] Li T, Anderson JJ (2009). The vitality model: a way to understand population survival and demographic heterogeneity. Theor Popul Biol.

[54] Wrigley-Field E (2014). Mortality deceleration and mortality selection: three unexpected implications of a simple model. Demography.

[55] Chen HY, Zajitschek F, Maklakov AA (2013). Why ageing stops: heterogeneity explains late-life mortality deceleration in nematodes. Biol Lett.

[56] Drapeau MD, Gass EK, Simison MD, Mueller LD, Rose MR (2000). Testing the heterogeneity theory of late-life mortality plateaus by using cohorts of Drosophila melanogaster. Exp Gerontol.

[57] Steinsaltz D (2005). Re-evaluating a test of the heterogeneity explanation for mortality plateaus. Exp Gerontol.

[58] Avraam D, Arnold-Gaille S, Jones D, Vasiev B (2014). Time-evolution of age-dependent mortality patterns in mathematical model of heterogeneous human population. Exp Gerontol.

[59] Black RE, Cousens S, Johnson HL, Lawn JE, Rudan I, Bassani DG, Jha P, Campbell H, Walker CF, Cibulskis R, Eisele T, Liu L, Mathers C (2010). and Child Health Epidemiology Reference Group of WHO and UNICEF. Global, regional, and national causes of child mortality in 2008: a systematic analysis. Lancet.

[60] Ahmad OB, Lopez AD, Inoue M (2000). The decline in child mortality: a reappraisal. Bull World Health Organ.

[61] Weibull W (1939). A statistical theory of the strength of materials.

[62] Weibull W (1951). A statistical distribution function of wide applicability. J Appl Mech.

[63] Le Bras H (2008). The nature of demography.

[64] Perks W (1932). On some experiments in the graduation of mortality statistics. Journal of the Institute of Actuaries (1886-1994).

[65] Kannisto V (1992).

[66] Beard RE, Brass W (1971). Some aspects of theories of mortality, cause of death analysis, forecasting and stochastic processes. Biological Aspects of Demography.

[67] Michaelis L, Menten ML (1913). Die kinetik der invertinwirkung. Biochem Z.

[68] Monod J (1949). The growth of bacterial cultures. Annu Rev Microbiol.

[69] Coale AJ, Kisker EE (1990). Defects in data on old-age mortality in the United States: new procedures for calculating mortality schedules and life tables at the highest ages. Asian Pac Popul Forum.

[70] Futuyma DJ (2013). Evolution.

[71] Hartl DL, Clark AG (2007). Principles of population genetics.

[72] Human Mortality Database

[73] Billo EJ (2007). Nonlinear regression using the Solver. Excel for Scientists and Engineers: Numerical Methods.

[74] Harris DC (1998). Nonlinear Least-Squares Curve Fitting with Microsoft Excel Solver. J Chem Educ.

[75] Schwarz G (1978). Estimating the Dimension of a Model.

[76] Hansen BE (2007). Least Squares Model Averaging. Econometrica.

[77] Priestley MB (1981). Spectral Analysis and Time Series.

[78] Gaille S (2012). Forecasting mortality: when academia meets practice. European Actuarial Journal.

[79] Gavrilova NS, Gavrilov LA (2015). Biodemography of old-age mortality in humans and rodents. J Gerontol A Biol Sci Med Sci.

[80] Hidalgo CA, Blumm N, Barabási A-L, Christakis NA (2009). A dynamic network approach for the study of human phenotypes. PLOS Comput Biol.

[81] Barabási A-L, Gulbahce N, Loscalzo J (2011). Network medicine: a network-based approach to human disease. Nat Rev Genet.

[82] Chmiel A, Klimek P, Thurner S (2014). Spreading of diseases through comorbidity networks across life and gender. New J Phys.

[83] GBD (2013). Mortality and Causes of Death Collaborators. Global, regional, and national age-sex specific all-cause and cause-specific mortality for 240 causes of death, 1990-2013: a systematic analysis for the Global Burden of Disease Study 2013. Lancet.

[84] Stearns SC, Byars SG, Govindaraju DR, Ewbank D (2010). Measuring selection in contemporary human populations. Nat Rev Genet.

[85] Pettay JE, Helle S, Jokela J, Lummaa V (2007). Natural selection on female life-history traits in relation to socio-economic class in pre-industrial human populations. PLoS One.

